# Multi-stage constant-current charging protocol for a high-energy-density pouch cell based on a 622NCM/graphite system

**DOI:** 10.1039/c9ra03629f

**Published:** 2019-07-10

**Authors:** Fuqiang An, Rui Zhang, Zhiguo Wei, Ping Li

**Affiliations:** Beijing University of Science and Technology No.30 Collage Road, Haidian District Beijing China liping@ustb.edu.cn; Shanxi Changzheng Power Technology Co., Ltd. Shanxi China; Idrivetech Automobile Co., Ltd. No. 2 Nanqi Road, ChangPing District Beijing China

## Abstract

A novel multi-stage-constant-current (MS-CC) charging protocol, which charges high-energy-density lithium-ion cells (LICs) at a faster rate, is presented herein. In this work, the 0–80% state of charge (SoC), according to the maximum charging rate, yields acceptable results for different SoCs, and the charging process is divided into three parts. Twelve groups of experiments are designed under the desired conditions of avoiding lithium plating and using a charging time of less than 36 min, and 1.5C constant current charging is used as a comparison experiment. The full pouch cells are dismantled, and the lithium deposition after 1.5C charging is more extensive than that after the MS-CC charging protocol. In addition, the capacity retention for 1.5C charging is 95.7%, while those for the 12 MS-CC charging protocol groups are within the range of 99.5–100.0% after the 300th cycle at 25 °C. When the temperature is 25 °C and 50 °C, the capacity retention of the 12 MS-CC charging protocol groups remains similar, but when the temperature drops to 10 °C, the capacity retention decreases except for the 2.0–1.5–0.9C and 1.8–1.5–0.9C groups. At the 510th cycle, the capacity retention of the 2.0–1.5–0.9C and 1.8–1.5–0.9C groups is 99.6% and 99.9%, respectively; the values of the other 10 groups are between 95.0% and 98.2%. The excellent electrochemical performances of the MS-CC charging protocol may be due to the minimal damage of cell materials caused by the step-type high-rate charging process; thus, the degree of polarization is small. Furthermore, compared with the conventional constant constant-current (CC) charging procedure, MS-CC charging greatly shortens the charging time.

## Abstract 2

A novel multi-stage-constant-current (MS-CC) charging protocol, which charges high-energy-density lithium-ion cells (LICs) at a faster rate, is presented herein.

## Graphical abstract

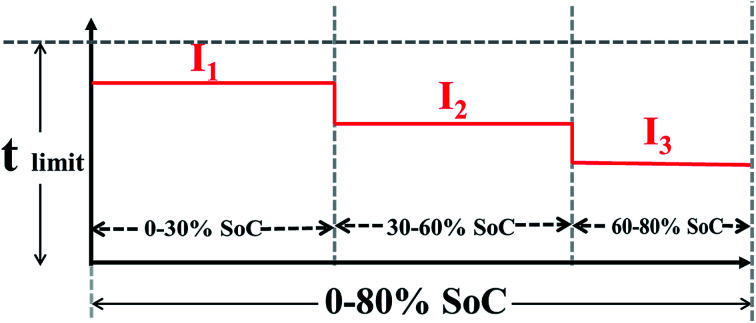

## 1 Introduction

With the rapid depletion of fossil fuels and severe environmental degradation, seeking alternative power sources is becoming imperative.^1,2^ Lithium-ion cells (LICs), one of the main energy storage devices, have become a topic of intense research focus because of their overwhelming advantages over other cells, such as higher energy density, longer cycle life and better eco-friendliness. Currently, LICs are widely applied to daily life, such as in portable electronic products, portable medical devices, and energy-saving industrial facilities.^3,4^ Additionally, LICs are the most promising power sources for electric vehicles (EVs) and plug-in hybrid electric vehicles (PHEVs), which are likely to be the next generation of green transportation.^5^ The mass commercialization of EVs and PHEVs may be largely reliant on whether researchers can improve the energy and power densities of LICs and lower their cost. However, higher energy density has a trade-off with higher power performance; considering the higher energy density requirements for the commercialization of LICs, it is critical to shorten the charging time in practice while ensuring the performance of the cells.

At present, the main charging protocol of commercial cells is the conventional constant current-constant voltage (CC–CV) charging protocol. The CC–CV charging protocol includes two continuous steps:; one step is the constant current charging stage, which lasts until the cell voltage reaches the preset value (4.1 or 4.2 V).^6,7^ The other step is when the voltage reaches the preset value: the system is switched to constant voltage charging, and the current will gradually decrease to another preset value.^6,7^ During the application of a constant voltage, the current slowly diminishes, which greatly extends the charging time.^6,7^ In addition, for the CC–CV protocol, using a high rate of charging for the LICs will lead to lithium plating in the high state of charge (SoC). When the rate of Li^+^ embedding in the surface of the anode material is faster than the diffusion inside the material, Li^+^ will accumulate on the surface of the anode electrode to form lithium metal.^8–10^ It is well known that lithium plating will affect the cycle performance and high/low-temperature performance in LICs and can lead to safety accidents. To lengthen the span life and restrain lithium plating on anode electrodes in LICs, a variety of charging protocols have been developed. For example, continuously varying current charging,^11,12^ pulse current charging,^13–15^ boost charging,^16^ constant power charging^17^ and multi-stage constant current charging^18,19^ protocols have been evaluated.

Sikha reported that the relationship between continuously varying current charging and time is a linear complex function, and the charging voltage of this method is always maintained under the cut-off voltage, thus realizing fast charging on the premise of not affecting the cell life.^12^ Pulse current charging, controlling the whole charging process by adjusting the current amplitude, pulse time, and pulse interval, can alleviate the polarization phenomenon and enhance the utilization rate of active materials, as well as improve the cycle performance.^14^ Judy's group used the Taguchi orthogonal array method to determine the optimal parameters of pulse charging. Compared with the CC–CV protocol, the energy and charge efficiencies of the pulse charge are increased by approximately 11.3% and 1.5%, respectively.^20^ The boost charging protocol refers to pre-charging a certain amount of charge with a large current in the initial charging stage (low SoC) and then switching to the CC–CV procedure. Notten *et al.* reported that the boost charging method can fulfil the requirement of rapidly charging LICs without causing any deterioration for the cell, which can be charged to 30% within 5 min under the empty state.^16^ Although the above rapid charging protocols shorten the charging time, the lithium plating caused by high-rate charging has not been specifically studied. It is universally known that under the conditions of low temperature, high rate and high SoC, metal lithium formation can readily occur at the surface of the anode electrode. Nuclear magnetic resonance (NMR) technology and charge–discharge curve analysis are used to detect metal lithium in LICs.^21–23^ However, these methods all have certain limitations. For example, NMR technology has a relatively higher level of demand for samples (extremely thin, suitable size), and charge–discharge curve analysis cannot detect lithium oxide on the surface at low or high SoC. In this work, the MS-CC charging protocol is adopted to achieve the required rapid charging of LICs. This method can not only shorten the charging time but also extraordinarily improve the cycling performance compared with the high-rate constant current charging protocol. Furthermore, to observe the phenomenon of metal lithium formation on the anode surface, the most intuitive disassembly technology is applied. Although this technology may be destructive to the cells, it is the most practical for commercial LICs. The test processes and electrochemical properties of the as-prepared lithium-ion full pouch cells are discussed in detail.

## 2 Experiment

### 2.1 Charging boundary determination

Twenty-six commercially available full pouch cells, which were sorted from forty-five cells based on capacity, mass ACR and OCV measurements, were charged between 0 and 80% SoC. These cells are mainly composed of cathode (611 NCM, LiNi_0.6_Co_0.6_Mn_0.2_O_2_), anode (graphite), separators and electrolyte. The electrode loading for the anode was 110 g m^−2^ (one side), with 95.1% active material, 3.7% binder (CMC/SBR) and 1.2% conductive carbon. The cathode loading was 190 g m^−2^ (one side), with 96.5% active material, 2% binder and 1.2% conductive carbon. The average energy density of these cells can be as high as 235 W h kg^−1^.

Combined with the actual charge requirement for EVs, charging more than 80% power in less than 40 min is acceptable. The SoC of 0–80% was separated into three parts, 0–30% SoC, 30–60% SoC and 60–80% SoC, and the metal lithium boundary of each part was evaluated (as shown in Table 1). The main reasons for dividing the 0–80% SoC into three parts are as follows: (1) low SoC charging with a high rate will not lead to deterioration of the cell materials; (2) a high SoC is prone to lithium plating, so this charging uses a small current; and (3) the charging time is less than 36 min. The maximum charging rates of each stage are determined by three experiments. In test 1, cells are charged with 2.8C (0–30% SoC), 2.5C (30–60% SoC) and 1.5C (60–80% SoC) and cycled five times in each stage. It is worth noting that in the second and third stages, the small current of 0.3C is used to charge the 30% and 60% SoC, respectively. To observe lithium deposition at the anode electrode interface, the cells are disassembled in the glove box after the cycling process. Test 1 shows that the three stages of the cells exhibit lithium deposition. Test 2 is designed to reduce the charge rate of three stages based on the results of test 1. However, after disassembly of the cell, poor results are obtained, similar to those in test 1. Therefore, the charge rates of the three stages are once again decreased to 2.2C, 1.9C and 0.9C for 0–30%, 30–60%, and 60–80% SoC, respectively. After 5 cycles, there is no grey metallic lithium on the surface of separators. Finally, the maximum charging rates of the three stages are finally ascertained to be 2.2C, 1.9C and 0.9C, respectively.

**Table 1:** Different SoC lithium plating boundary

No.	Type SoC	0–30%	30–60%	60–80%
Test 1	Charge rate/C	2.8C	2.5C	1.5C
	Interface	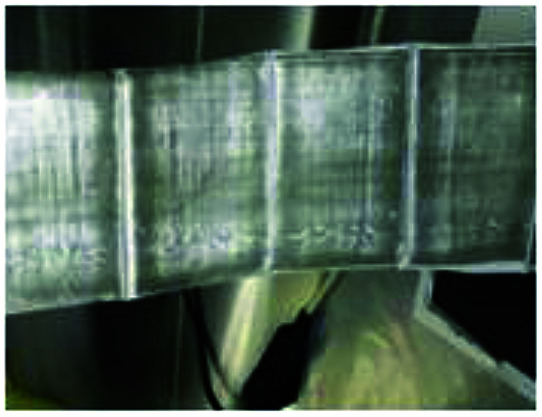	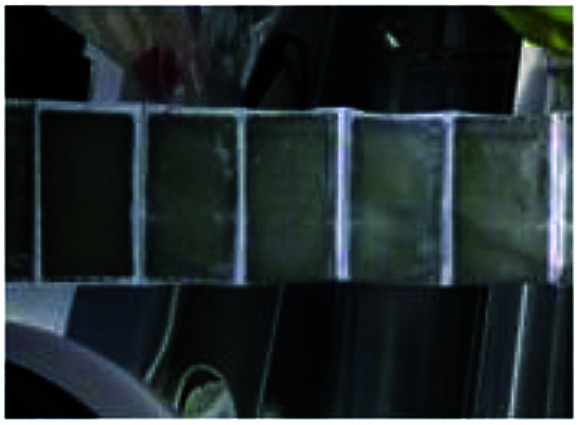	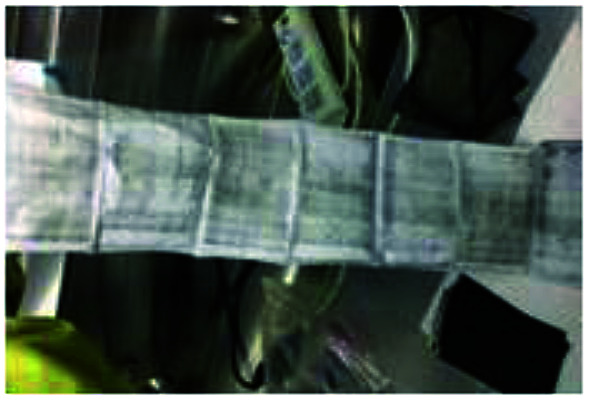
Test 2	Charge rate/C	2.5C	2.2C	1.2C
	Interface	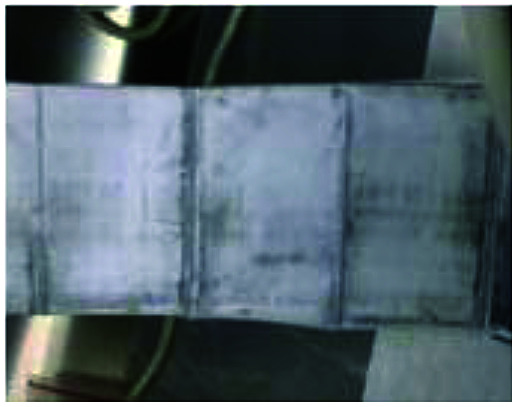	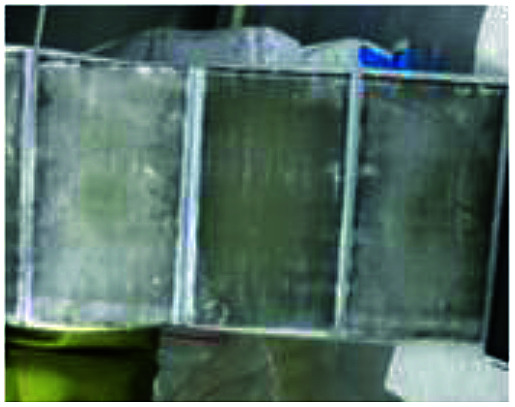	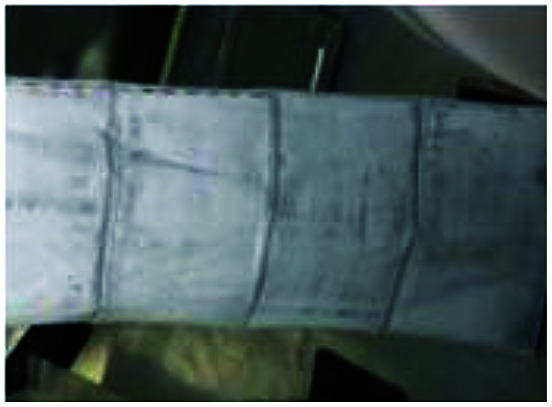
Test 3	Charge rate/C	2.2C	1.9C	0.9C
	Interface	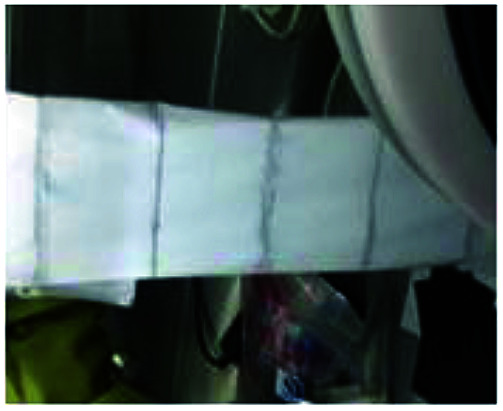	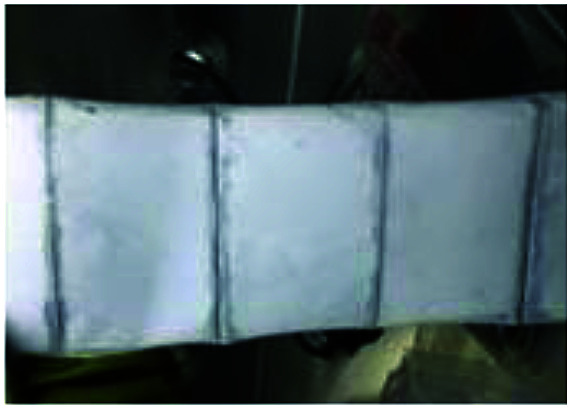	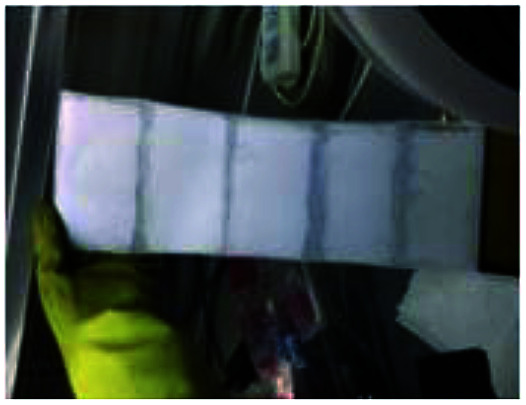

### 2.2 Cycle performance

**Fig. 1:**
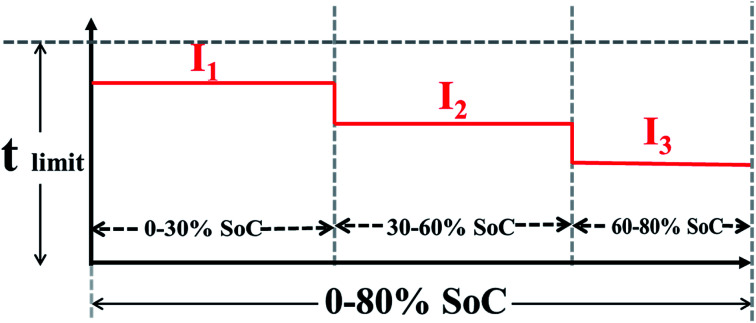
Schematic illustrates the charging process of multi-stage constant current charging protocol.

The galvanostatic charge–discharge tests were performed on a Neware cell test system (Shenzhen, China) at different current densities between 2.5 and 4.25 V (*vs.* Li^+^/Li). On the basis of charging boundary conditions, 12 groups of rapid charging schemes are established, and 1.5C continuous constant current charging is used as a reference experiment (two cells in each group). These charging schemes are shown in Table 2, and the test procedure of the MS-CC charging is schematically shown in Fig. 1. During the testing process, each stage is separated based on time, and an under-voltage and over-voltage protection program is set. To standardize all the cells, capacity calibration is performed before the rapid charging test. These cells are charged at 0.5C to 4.25 V and then discharged at 1C to 2.5 V for three cycles. To further eliminate the interference of temperature factors on the experimental results, the temperature changes in one cycle of the 13-group charging scheme are monitored during charging and discharging processes and then used to determine the shelving time of the charge–discharge process. During the process, the thermocouple is positioned in the middle of the cells, and its accuracy is 0.5 °C. After charging, G1–G12 take 7 min to reach room temperature, G13 takes 15 min to drop to room temperature, and all cells need to be cooled room temperature for 10 min after discharging. The temperature changes of charging at different rates under different SoCs are listed in Table 3.

**Table 2:** 13 groups charging scheme of 0 to 80% SoC

Group	0–30% SoC	30–60% SoC	60–80% SoC	Charge time/min
1	2.2C	1.9C	0.9C	31
2	2.2C	1.9C	0.7C	34.8
3	2.2C	1.7C	0.9C	32.1
4	2.2C	1.7C	0.7C	35.9
5	2.2C	1.5C	0.9C	33.5
6	2C	1.9C	0.9C	31.8
7	2C	1.9C	0.7C	35.61
8	2C	1.7C	0.9C	32.9
9	2C	1.5C	0.9C	34.3
10	1.8C	1.9C	0.9C	32.8
11	1.8C	1.7C	0.9C	33.9
12	1.8C	1.5C	0.9C	35.3
13	1.5C	1.5C	1.5C	32

**Table 3:** The temperature changes of 13 groups charging scheme of charging–discharging

Group	Pre-test temperature °C	Charging °C						Discharging °C	
		30% SoC	Δ*T*	60% SoC	Δ*T*	80% SoC	Δ*T*	Temperature	Δ*T*
G1: 2.2–1.9–0.9	23.8	26.2	2.4	26.4	2.6	24.8	1.00	25.4	1.6
G2: 2.2–1.9–0.7	23.6	26.4	2.8	26.6	3	24.3	0.70	25.6	2
G3: 2.2–1.7–0.9	23.8	26.5	2.7	26.4	2.6	24.7	0.90	25.9	2.1
G4: 2.2–1.7–0.7	23.7	26.5	2.8	26.2	2.5	24.3	0.60	25.6	1.9
G5: 2.2–1.5–0.9	23.9	26	2.1	25.9	2	24.6	0.70	25.2	1.3
G6: 2–1.9–0.9	23.7	26.1	2.4	26.5	2.8	24.4	0.70	25.2	1.5
G7: 2–1.9–0.7	23.7	26	2.3	26.7	3	24.3	0.60	25.8	2.1
G8: 2–1.7–0.9	23.3	25.7	2.4	26	2.7	24.5	1.20	25.4	2.1
G9: 2–1.5–0.9	23.4	25.7	2.3	25.4	2	24.4	1.00	25.4	2
G10: 1.8–1.9–0.9	24	25.9	1.9	26.6	2.6	25	1.00	25.8	1.8
G11: 1.8–1.7–0.9	23.7	26	2.3	26.7	3	24.8	1.10	25.8	2.1
G12: 1.8–1.5–0.9	23.7	25.6	1.9	25.6	1.9	24.6	0.90	25.6	1.9
G13: 1.5–1.5–1.5	23.9	25.7	1.8	26.2	2.3	26.5	2.60	26.4	2.1

After the above pre-test, the 13 groups of cells are cycled at 25 °C, 50 °C and 10 °C with designed charge protocols, and the discharge rate is 1C for all the cells.

### 2.3 Polarization characterization

In an increment of every 100 cycles, the cycling tests are terminated, and the cells are brought to the fully charged state using a 0.5C CC–CV protocol, followed by impedance and ac resistance measurements. To explore the polarization characterization of the 13 charging scheme groups, electrochemical impedance spectroscopy (EIS) measurements were performed on an electrochemical workstation (VMP3, France) with an AC voltage of 5 mV amplitude in the frequency range of 10^5^ to 10^−2^ Hz.

The differential voltage (d*Q*/d*V*) is used to differentiate the changes in the redox peaks. Fresh and aged cells were measured using the VMP3 system with 0.2C charge and discharge, and the data acquisition frequency was 1 s.

### 2.4 Analysis for post-mortem state

To gain insight into the lithium plating phenomenon of the cells in the post-mortem state, the full pouch cells were disassembled in an argon-filled glove box with water and oxygen concentrations below 1 ppm.

## 3 Results and discussion

**Fig. 2:**
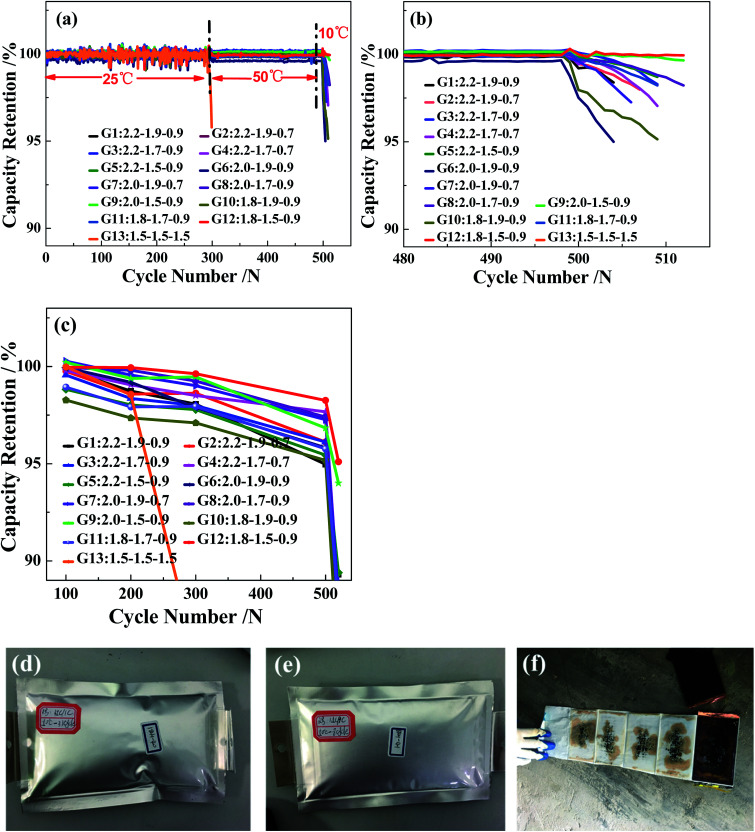
The different temperatures of 13 groups charging scheme (a and b) the capacity retention, (c) the capacity calibration, 1.5C constant current chargeable cells after 300 cycles (d and e), and disassemble appearance image (f).

To investigate the electrochemical properties of the 13 charging scheme groups, the cycle performance and capacity recovery capabilities are tested at a voltage range of 2.5–4.25 V. Fig. 2a and b show the capacity retention of the 13 charging scheme groups under different temperatures. The reason for applying different cycle temperatures in this work is to quickly screen out the optimal rapid charging combination at 0–80% SoC. It can be clearly seen from Fig. 2a that the capacity retention of multi-stage current charging slightly varies at 25 °C and 50 °C, but when charging at 1.5C constant current (G13), the capacity retention of the 300th cycle is seriously attenuated at 25 °C. To make matters worse, the cells of the 1.5C constant current have a flatulence phenomenon after 300 cycles (Fig. 2d and e). After disassembling the 1.5C constant current charging cell, many impurities are observed on the anode electrode and separator, possibly due to the continuous charging at a high rate, which resulted in the production of metal lithium and reacted with the electrolyte (Fig. 2f). This result proves that the MS-CC charging protocol is more suitable for high-rate rapid charging than single high-rate constant current charging. The state of the charging cycles at 10 °C is shown in Fig. 2b. The capacity retention of all cells shows a downward trend, but the extents of decline for 2.0–1.5–0.9C (G9) and 1.8–1.5–0.9C (G12) are the smallest relative to the other 10 groups. The capacity retention of G9 and G12 are 99.6% and 99.9%, respectively, whereas those of the other 10 groups are between 95.0% and 98.2% at the 510th cycle. The capacity retention of the capacity calibration for each 100 cycles of the 13 charging scheme groups is presented in Fig. 2c, in which the capacity retention of all cells gradually decreases as the number of cycles increases. Comparing the capacity retention of all cells, the decline rate of G13 is fastest, with 85.0%, and those of G9 and G12 are the slowest (94.0 and 95.1%, respectively). The above results further demonstrate that the MS-CC charging protocol is better than high-rate constant current charging for the rapid charging of LICs. In addition, after 36 min, groups G9 and G12 have the best electrochemistry performance compared to the other 10 groups of rapid charging schemes, proving that the combination of charging rates is equally important.

**Fig. 3:**
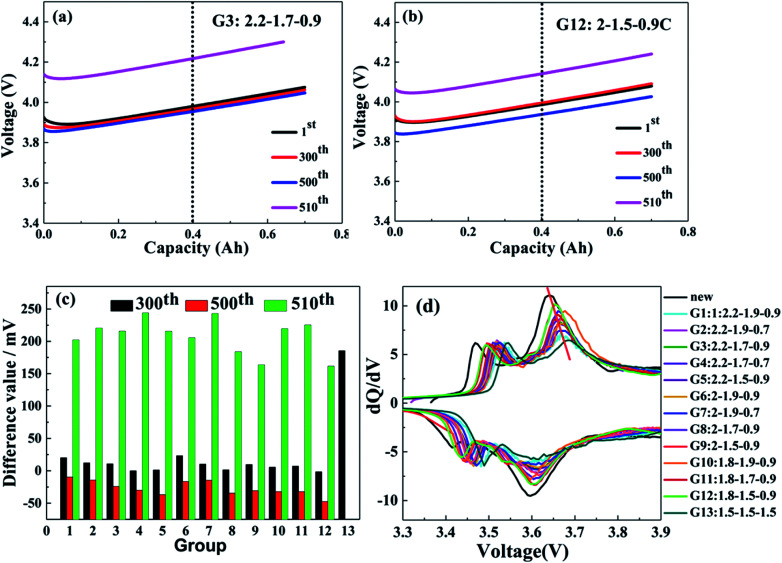
60–80% charging curves of G12 (a) and G3 (b), the difference value of 13 groups charging scheme (c), d*Q*/d*V* curves of 13 groups charging scheme and new cell (d).

The effect of the charging protocol on cell polarization is displayed in Fig. 3. Fig. 3a–c show the charge curves of the G3 and G12 groups under 60–80% SoC at the 1st, 300th, 500th and 510th cycles. The polarization degree of cells is expressed by the voltage of the 300th, 500th and 510th cycles (corresponding to the voltage charged to 0.4 A h) minus the voltage of the 1st cycle. It is calculated that the difference values of the 300th, 500th and 510th cycles of G12 are −1.5 mV, −47.4 mV and 161.5 mV, respectively, which are much lower than those of G3, with 10.8 mV (300th), −24.4 mV (500th) and 202.2 mV (510th). In addition, it is clear that the difference values of the 300th, 500th and 510th cycles of G9 and G12 are lower than those of the other groups; the difference value of the 300th cycle of G13 is the largest, which proves that the MS-CC charging protocol is less destructive to cell materials and that the polarization degrees of G9 and G12 are the lowest. The differential voltage (d*Q*/d*V*) curves can elucidate some of the locations and types of fade mechanisms in LICs.^24,25^ The peaks on the d*Q*/d*V* curves originate from phase transitions, and the translation of the peak position represents the change in the cell internal resistance.^26^Fig. 3d shows the d*Q*/d*V* curves of the 13 charging scheme groups and a new cell, in which the curve of the new cell has two oxidation peaks and two reduction peaks. Because this experiment is a full pouch cell test, the curve shows the common peaks of the cathode and anode materials. The curves of the other 13 groups are similar to those of the new cell, indicating that there is no phase transition during the cycle. As the benchmark curve of the new cell, the oxidation peaks of G9 and G12 shift to the right the least distance, illustrating that their internal resistance is smallest in the 13 charging scheme groups. In addition, the peak intensity reflects the content of active materials, and the peak intensity will weaken with the loss of active materials.^27^ It can be seen from Fig. 3d that the peak intensity of the 13 charging scheme groups is lower than that of the new cell, but G13 has the lowest peak intensity relative to the other groups, which indicates that high-rate constant current charging will lead to additional loss of active materials. Furthermore, the peak strengths of G9 and G12 are the strongest among the 12 rapid charging scheme groups, suggesting that the charging rate of these two groups is beneficial to the rapid charging of LICs.

**Fig. 4:**
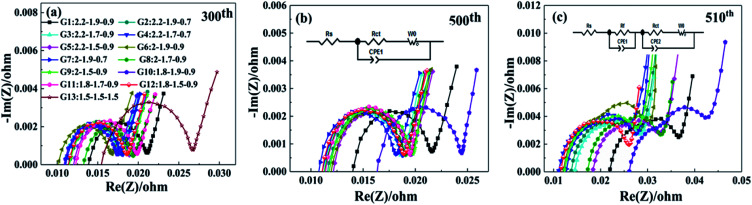
The EIS curves the 13 groups charging scheme different cycle numbers of 300th (a), 500th (b) and 510th (c).

To investigate the effects of the 13 charging scheme groups on the cell electrochemical performance, EIS measurements were carried out. Fig. 4 shows the Nyquist plots of the 13 charging scheme groups after the 300th, 500th and 510th cycles under 50% SoC. As seen in Fig. 4a and b, all the impedance spectra are composed of a single semicircle in the high-frequency region and an inclined line in the low-frequency region.^28–30^ Generally, the semicircle is closely related to the charge transfer resistance (*R*_ct_), which reflects the electrode reaction kinetics, and the inclined line represents the Warburg impedance (*Z*_w_), which is caused by the diffusion of lithium ions into the active material.^31^ The intercept of the semicircle with the *Z*′-axis in the high-frequency region refers to unremunerative ohmic resistance (*R*_s_), which includes the resistance of the electrolyte, Li metal anode and Al foil current collector.^32^ Based on the abovementioned, the equivalent circuit for the electrodes is shown in Fig. 4b and c, and the related simulated electrochemical parameters are tabulated in Table 4. As shown in Fig. 4a, the G13 cell delivers a *R*_ct_ value of 0.0267 Ω, which is significantly higher than that for the other 12 groups, ranging from 0.0166 to 0.0211 Ω; this result shows that the multi-stage charging protocol attains a smaller electrochemical polarization. After 510 cycles, there are two semicircles that appear in the Nyquist plots of the samples (Fig. 4c). The semicircle located in the high-frequency region is assigned to the impedance resulting from the generation of SEI films (*R*_f_), while another neighbouring semicircle in the mid-frequency region is ascribed to the *R*_ct_.^33,34^ It can be observed from Fig. 4b and c that the *R*_ct_ values of G9 and G12 increased slowly as the number of cycles increased compared with the other 10 groups. G9 and G12 possess lower *R*_ct_ values, which may be attributed to the fact that the current combination of these two groups does less damage to the cell materials during the charging process.

**Table 4:** Impedance indexes simulated from the equivalent circuits

Cycle number	Resistance	G1	G2	G3	G4	G5	G6	G7	G8	G9	G10	G11	G12	G13
300	*R*_s_ (mΩ)	13.9	11.5	11.0	11.2	12.1	10.2	11.1	13.3	11.5	11.0	12.4	11.5	15.5
	*R*_ct_ (mΩ)	21.0	18.6	17.7	18.0	18.9	16.6	17.9	19.3	18.7	17.3	19.7	18.5	26.7
500	*R*_s_ (mΩ)	14.0	11.7	11.4	11.1	12.0	12.0	10.9	11.3	11.7	16.4	11.6	11.1	—
	*R*_ct_ (mΩ)	21.7	19.0	18.7	18.8	19.5	19.4	18.2	19.0	19.7	24.4	19.4	18.8	—
510	*R*_s_ (mΩ)	22.0	13.7	14.5	11.0	18.4	12.1	12.7	17.2	12.3	26.1	12.8	11.6	—
	*R*_f_ (mΩ)	29.7	22.6	23.3	18.6	27.2	20.4	20.2	23.6	20.6	31.9	24.1	19.4	—
	*R*_ct_ (mΩ)	36.5	27.9	28.4	26.3	32.8	29.6	27.7	32.7	27.9	42.2	28.6	26.0	—

**Fig. 5:**
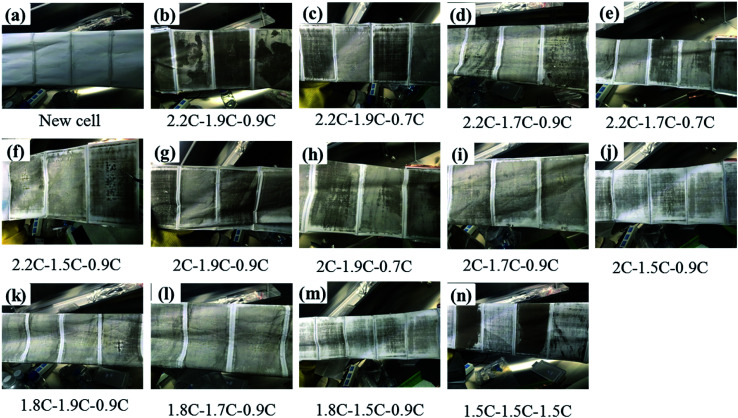
Graphic of separators' surface for 13 groups experiments after cycled with different charge rates.

To realize the rapid charging technology of LICs, it is necessary to determine the maximum current that the cells can withstand under different SoC conditions (the lithium plating boundary). Comparisons of metal lithium formation at different charging rates are made by observing the surface of all the cell separators. As shown in Fig. 5, metal lithium (grey part) exists on the surface of all the separators except those of the new cells (which have only been calibrated for capacity). It is observed that the lithium plating degree shown in Fig. 5j (G9) and *m* (G12) is clearly lower than that of the other groups (Fig. 5b–i, k, l and n). It is obvious that carbon is bound to the surface of the separator for the b, c and n series.

## 4 Conclusions

The key factor in realizing the rapid charging technology of LICs is to circumvent the phenomenon of lithium plating. To overcome this problem, a MS-CC charging protocol for higher energy density (235 W h kg^−1^) pouch cells with a 611 NCM/graphite system was carried out in this work. To quickly screen out the optimal combination of charging rate at 0–80% SoC, the cells were tested at 25 °C, 50 °C and 10 °C. By comparing the 13 charging scheme groups, it is concluded that G9 (2.0–1.5–0.9C) and G12 (1.8–1.5–0.9C) have the best electrochemical performance after 510 cycles. The interfacial lithium plating at the separator surface is the most minor for these two groups compared with the other 11 groups, indicating that MS-CC charging is more suitable for high-rate fast charging. In addition, the capacity retention values of G9 and G12 are 99.6% and 99.9%, while those of the other 10 groups are in the range of 95.0–98.2% at the 510th cycle. Similarly, G9 and G12 have the lowest polarization, which is expressed by evaluating the difference values of the same point during the cycle at 60–80% SoC. The results indicate that these favourable electrochemical properties can be mainly attributed to the suitable combination of charging rate at different SoCs, which can decrease polarization and restrain the precipitation of lithium metal during the charging process. In addition, the MS-CC charging not only improves the cycle performance but can also shorten the charging time.

## Conflicts of interest

There are no conflicts to declare.

## Supplementary Material

## References

[cit1] Wang F. X., Wu X., Li C. (2016). *et al.*, Nanostructured positive electrode materials for post-lithium ion batteries. Energy Environ. Sci..

[cit2] Chu S., Cui Y., Liu N. (2017). The path towards sustainable energy. Nat. Mater..

[cit3] Wu S. F., Wang W. X., Li M. C., Cao L. J., Lu F., Yang M. Y., Wang Z. Y., Shi Y., Nan B., Yu S. C., Sun Z. F., Liu Y., Lu Z. G. (2016). Highly durable organic electrode for sodium-ion batteries via a stabilized α-C radical intermediate. Nat. Commun..

[cit4] Cao D. P., Yin C. L., Shi D. R., Fu Z. W., Zhang J. C., Li C. L. (2017). Cubic Perovskite Fluoride as Open Framework Cathode for Na-Ion Batteries. Adv. Funct. Mater..

[cit5] Hy S., Liu H., Zhang M., Qian D., Hwang B.-J., Meng Y. S. (2016). Performance and Design Considerations for Lithium Excess Layered Oxide Positive Electrode Materials for Lithium Ion Batteries. Energy Environ. Sci..

[cit6] Xu M., Wang R., Reichman B., Wang X. (2018). Modeling the effect of two-stage fast charging protocol on thermal behavior and charging energy efficiency of lithium-ion batteries. J. Energy Storage.

[cit7] Spingler F. B., Wittmann W., Sturm J., Rieger B., Jossen A. (2018). Optimum fast charging of lithium-ion pouch cells based on local volume expansion criteria. J. Power Sources.

[cit8] Gao Y., Jiang J., Zhang C., Zhang W., Ma Z., Jiang Y. (2017). Lithium-ion battery aging mechanisms and life model under different charging stresses. J. Power Sources.

[cit9] Bolsinger C., Birke K. P. (2019). Effect of different cooling configurations on thermal gradients inside cylindrical battery cells. J. Energy Storage.

[cit10] Zeng Z., Liang W.-I., Liao H.-G., Xin H. L., Chu Y.-H., Zheng H. (2014). Visualization of Electrode-Electrolyte Interfaces in LiPF_6_/EC/DEC Electrolyte for Lithium Ion Batteries via in Situ TEM. Nano Lett..

[cit11] Chung S. K., Andriiko A. A., Mon'Ko A. P. (1999). On charge conditions for Li-ion and other secondary lithium batteries with solid intercalation electrodes. J. Power Sources.

[cit12] Sikha G., Ramadass P., Haran B. S. (2003). Comparison of the capacity fade of Sony US 18650 cells charged with different protocols. J. Power Sources.

[cit13] Hasan M. F., Chen C., Shaffer C. E. (2015). Analysis of the implications of rapid charging on lithium-ion battery performance. J. Electrochem. Soc. India.

[cit14] Li J., Murphy E., Winnick J. (2001). The effects of pulse charging on cycling characteristics of commercial lithium-ion batteries. J. Power Sources.

[cit15] Zhao X. W., Zhang G. Y., Yang L. (2001). A new charging mode of Li-ion batteries with LiFePO_4_/C composites under low temperature. J. Therm. Anal. Calorim..

[cit16] Notten P., Veld J., Beek J. (2005). Boostcharging Li-ion batteries: A challenging new charging concept. J. Power Sources.

[cit17] Zhang S. S. (2006). The effect of the charging protocol on the cycle life of a Li-ion battery. J. Power Sources.

[cit18] Liu Y. H., Teng J. H., Lin Y. C. (2005). Search for an optimal rapid charging pattern for lithium-ion batteries using ant colony system algorithm. IEEE Trans. Ind. Electron..

[cit19] Liu Y., Hsieh C., Luo Y. (2011). Search for an optimal five-step charging pattern for Li-ion batteries using consecutive orthogonal arrays. IEEE Trans. Energy Convers..

[cit20] Amanor-Boadu J. M., Guiseppi-Elie A., Sánchez-Sinencio E. (2018). Search for Optimal Pulse Charging Parameters for Li-ion Polymer Batteries Using Taguchi Orthogonal Arrays. IEEE Trans. Ind. Electron..

[cit21] Arai J., Okada Y., Sugiyama T., Izuka M., Gotoh K., Takeda K. (2014). In situ Solid State 7Li NMR Observation of Lithium Metal Deposition during Overcharge in Lithium Ion Battery. ECS Trans..

[cit22] Petzl M., Danzer M. A. (2014). Nondestructive detection, characterization, and quantification of lithium plating in commercial lithium-ion batteries. J. Power Sources.

[cit23] Waldmann T., Wohlfahrt-Mehrens M. (2017). Effects of rest time after Li plating on safety behavior—ARC tests with commercial high-energy 18650 Li-ion cells. Electrochim. Acta.

[cit24] Li X., Jiang J., Wang L. Y., Chen D., Zhang Y., Zhang C. (2016). A capacity model based on charging process for state of health estimation of lithium ion batteries. Appl. Energy.

[cit25] Wang G., Yi L., Yu R., Wang X., Wang Y., Liu Z., Wu B., Liu M., Zhang X., Yang X., Xiong X., Liu M. (2017). Li_1.2_Ni_0.13_Co_0.13_Mn_0.54_O_2_ with Controllable Morphology and Size for High Performance Lithium-Ion Batteries. ACS Appl. Mater. Interfaces.

[cit26] Ando K., Matsuda T., Imamura D. (2018). Degradation diagnosis of lithium-ion batteries with a LiNi_0.5_Co_0.2_Mn_0.3_O_2_ and LiMn_2_O_4_ blended cathode using dV/dQ curve analysis. J. Power Sources.

[cit27] Liu S., Xiong L., He C. (2014). Long cycle life lithium ion battery with lithium nickel cobalt manganese oxide (NCM) cathode. J. Power Sources.

[cit28] Zhang R., Wang X. Y., Wei S. Y., Wang X., Liu M., Hu H. (2017). Iron fluoride microspheres by titanium dioxide surface modification as high capacity cathode of Li-ion batteries. J. Alloys Compd..

[cit29] Wang G., Yi L. L., Yu R. Z., Wang X. Y., Wang Y., Liu Z. S., Wu B., Liu M., Zhang X. H., Yang X. K., Xiong X. H., Liu M. L. (2017). Li_1.2_Ni_0.13_Co_0.13_Mn_0.54_O_2_ with Controllable Morphology and Size for High Performance Lithium-Ion Batteries. ACS Appl. Mater. Interfaces.

[cit30] Huang X., Yu H., Chen J., Lu Z. Y., Yazami R., Hng H. H. (2014). Ultrahigh Rate Capabilities of Lithium-Ion Batteries from 3D Ordered Hierarchically Porous Electrodes with Entrapped Active Nanoparticles Configuration. Adv. Mater..

[cit31] Ruffo R., Hong S. S., Chan C. K., Huggins R. A., Cui Y. (2015). Impedance analysis of silicon nanowire lithium ion battery anodes. J. Phys. Chem. C.

[cit32] Li C., Han X. P., Cheng F. Y., Hu Y. X., Chen C. C., Chen J. (2015). Phase and composition controllable synthesis of cobalt manganese spinel nanoparticles towards efficient oxygen electrocatalysis. Nat. Commun..

[cit33] Zheng L., Liu L., Zhou X., Guo Y. (2014). An Electrochemical Impedance Spectroscopy (EIS) Study of Zn-doped Li (Ni_1/3_Co_1/3_Mn_1/3_) O_2_ Cathode Materials in the First Delithiation Process. Adv. Mater. Res..

[cit34] Lu B., Ma B., Deng X., Wu B., Wu Z., Luo J., Wang X., Chen G. (2018). Dual Stabilized Architecture of Hollow Si@TiO_2_@C Nanospheres as Anode of High-Performance Li-Ion Battery. Chem. Eng. J..

